# Use of monthly extended-release risperidone injection in schizophrenia: clinical experience

**DOI:** 10.1192/j.eurpsy.2024.451

**Published:** 2024-08-27

**Authors:** E. de la Fuente Ruiz, M. D. C. Blasco Fresco, C. Álvarez Sanagustín, L. T. Durán Sandoval

**Affiliations:** ^1^Psychiatry, Hospital Universitario Miguel Servet; ^2^Department of Medicine, Psychiatry and Dermatology, University of Zaragoza; ^3^Family and Community Medicine, Hospital Universitario Miguel Servet, Zaragoza, Spain

## Abstract

**Introduction:**

Monthly extended-release injectable risperidone is the new antipsychotic formulation of risperidone available in doses of 75 mg and 100 mg, approved for the treatment of schizophrenia. It contains microcrystals of risperidone that are deposited following intramuscular injection. A fraction of the active ingredient of risperidone is already solubilized and rapidly enters the bloodstream, providing plasma levels similar to oral risperidone on the first day. The microcrystals continue to release risperidone steadily over a period of 4 weeks. No oral supplementation or loading doses are required.

**Objectives:**

The objective of this study is to demonstrate the effectiveness of treatment with monthly extended-release injectable risperidone in patients with schizophrenia who are followed up as outpatients from the Mental Health Center. The study aims to show that this treatment improves symptoms associated with schizophrenia, leading to an enhancement in the quality of life for these patients.

**Methods:**

Analysis and evaluation were conducted on 9 patients diagnosed with Paranoid Schizophrenia and treated with monthly extended-release injectable risperidone from a Mental Health Unit and the Hospital Emergency System during the months of January to April 2023. Among the nine patients, six were previously on oral risperidone treatment exceeding 4 mg, and three were on doses less than 4 mg. The first group received a monthly injectable dose of 100 mg of risperidone, while the second group received 75 mg.

**Results:**

All nine patients showed improvement in positive and anxious symptomatology. Seven of them exhibited improvement in affective and cognitive profiles. None of the patients experienced significant metabolic alterations, and only one of them reported akathisia as a side effect. Furthermore, all patients improved their sleep patterns, and the seven who had behavioral disturbances with a tendency towards aggression no longer exhibited these behaviors.

**Conclusions:**

Monthly extended-release injectable risperidone is beneficial in reducing positive and affective symptoms in patients with schizophrenia. It also improves anxious, cognitive, and behavioral symptomatology. It is considered effective, safe, and well-tolerated for long-term treatment of this disease, regardless of its initial severity. Therefore, it is advisable to consider it as the first therapeutic option in patients with schizophrenia who have responded well to oral risperidone previously.

**Disclosure of Interest:**

None Declared

